# Uvular Necrosis After Shoulder Surgery: A Report of Three Cases

**DOI:** 10.7759/cureus.14233

**Published:** 2021-03-31

**Authors:** Michelle Xiao, David I Kaufman, Geoffrey D Abrams

**Affiliations:** 1 Orthopaedic Surgery, Stanford University School of Medicine, Stanford, USA; 2 Anesthesiology, Perioperative and Pain Medicine, Stanford University School of Medicine, Stanford, USA

**Keywords:** uvular necrosis, shoulder surgery, anesthesia, intubation, ett, lma, sore throat

## Abstract

Uvular necrosis is a rare postoperative complication that can manifest from endotracheal tube intubation or laryngeal mask airway placement resulting in compression and restriction of blood flow to the uvula. This report describes three patients who underwent outpatient shoulder surgery under general anesthesia and were subsequently diagnosed with uvular necrosis. Their symptoms included persistent sore throat, dysphagia, odynophagia, and foreign body sensation, with swelling and white exudate on the uvular tip. All three patients were treated conservatively and had complete symptom resolution. While symptoms from uvular necrosis typically self-resolve within two weeks, it is important to recognize the condition and risk factors because patients may benefit from reassurance and conservative treatment.

## Introduction

Postoperative uvular necrosis is a rare complication that can result from securing the airway through the endotracheal tube (ETT) or laryngeal mask airway (LMA) [1-5]. Not much is known about the etiology of uvular necrosis, although it is thought to be caused by rigid compression or direct suction compromising blood supply to the uvula. Limited case reports have documented that patients with postoperative uvular necrosis may present with persistent sore throat, dysphagia, foreign body sensation, or gagging, and the tip of the uvula can be ischemic, inflamed, elongated, and covered with white exudate [6]. Although general anesthesia is administered for many shoulder procedures, uvular necrosis has not been well described in the orthopedic literature. Here, we present case reports of three varsity athletes at our institution who underwent outpatient shoulder surgery during a four-month period and were subsequently diagnosed with uvular necrosis. The patients were informed that data concerning their cases would be submitted for publication, and they provided informed consent.

## Case presentation

Case 1

**Figure 1:**
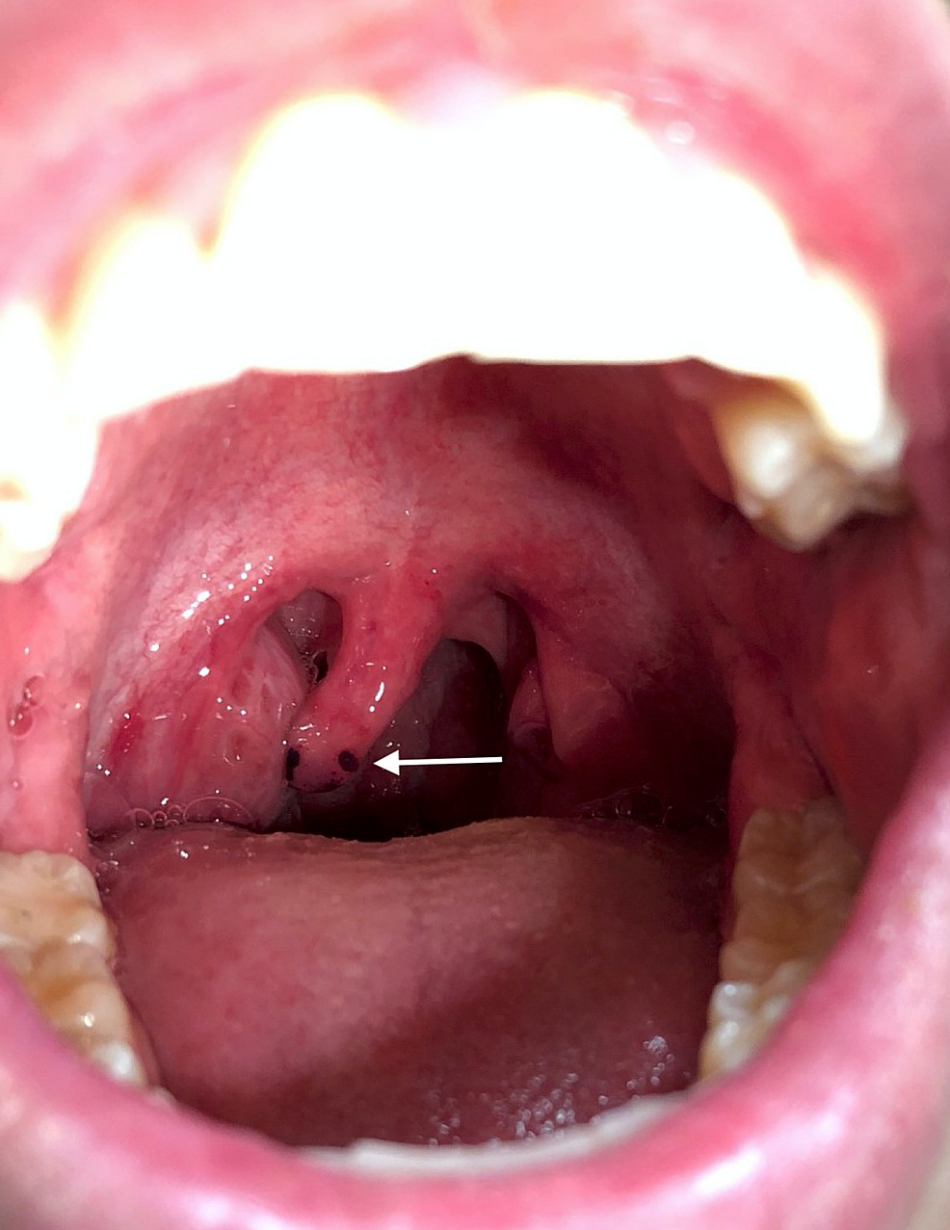
Oropharynx of the patient on postoperative day one demonstrating discoloration at the uvular tip.

A 20-year-old male football player weighing 87.1 kg with no relevant past medical history underwent a right shoulder arthroscopy, synovectomy, and posterior labral repair in the lateral decubitus position under general anesthesia. His American Society of Anesthesiologists (ASA) status was ASA I. Intubation was performed easily for a Mallampati class I airway using a size 5 LMA. The procedure lasted 133 minutes, and the patient tolerated the surgery well. The following day, the patient presented to the clinic with a sore throat but could tolerate food and water without issue. The patient denied any fever, vomiting, or difficulty breathing. On examination, we observed an elongated, erythematous uvula with spotty areas and discoloration at the tip (Figure 1).

The airway was not compromised. He was treated symptomatically with non-steroidal anti-inflammatory drugs as needed. The patient’s symptoms self-resolved within 14 days.

Case 2

**Figure 2:**
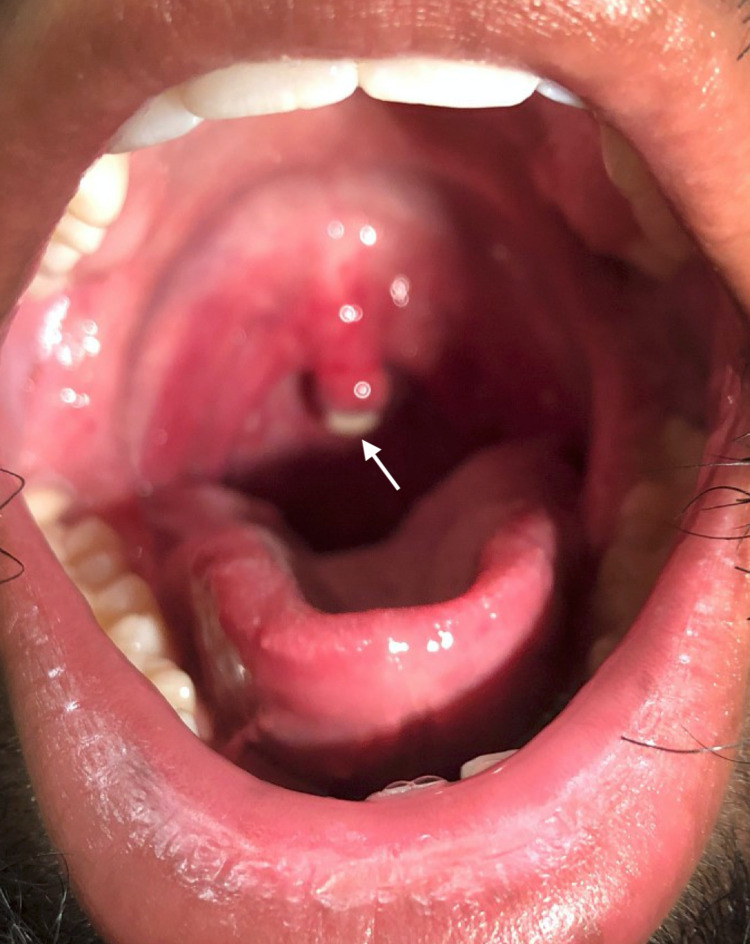
Oropharynx of the patient on postoperative day four demonstrating uvular necrosis.

A 22-year-old male football player (97.1 kg, ASA I) with no relevant past medical history underwent a left shoulder open reduction and internal fixation of a glenoid fracture and open capsulorrhaphy in the beach chair position under general anesthesia. Intubation was achieved on the first attempt by direct laryngoscopy using a size 3 Macintosh blade. Placement of the size 7.0 oral cuffed ETT was uneventful for a Mallampati class I airway. The procedure lasted 230 minutes, and the patient tolerated the surgery well. Two days later, the patient called our urgent care nurse complaining of throat pain, swelling, and a white spot at the tip of the uvula that had been present since surgery, causing him difficulty with talking and swallowing foods. He was followed up in the clinic on postoperative day four stating his throat pain was a four out of ten but improving. The patient denied any fever, vomiting, or difficulty breathing. On examination, we identified ecchymosis and necrosis to a significant portion of the uvular tip (Figure 2).

The airway was not compromised. The patient was treated with throat lozenges and magic mouthwash containing diphenhydramine, viscous lidocaine suspension, and aluminum hydroxide/magnesium hydroxide for pain control and was advised to maintain a soft food diet. The area of uvular necrosis subsequently fell off on postoperative day six and symptoms self-resolved soon after.

Case 3

**Figure 3:**
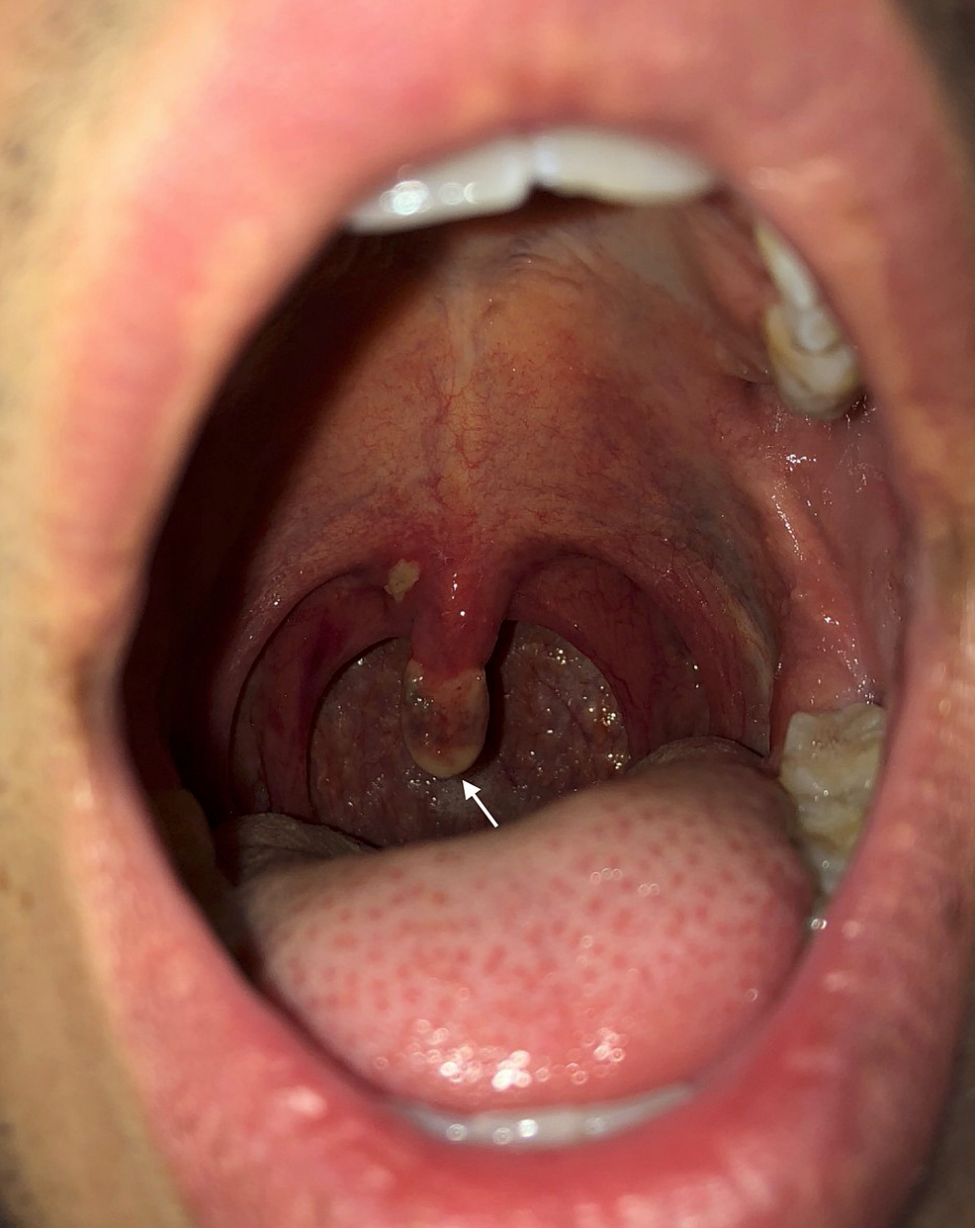
Oropharynx of the patient on postoperative day two demonstrating an elongated and necrotic uvula.

A 21-year-old male gymnast (71.7 kg, ASA II) underwent a right shoulder arthroscopy with intra-articular debridement, subacromial decompression, and mini open distal clavicle excision in the beach chair position under general anesthesia. His past medical history included postoperative nausea and vomiting following right anterior cruciate ligament reconstruction and a heterozygotic prothrombin gene mutation. Intubation was achieved on the first attempt by direct laryngoscopy using a size 3 Macintosh blade. Placement of the size 7.0 oral cuffed ETT was uneventful for a Mallampati grade II airway. The procedure lasted 113 minutes, and the patient tolerated the surgery well. The following day, the patient called our triage nurse complaining of sensitivity to heat, pain, and a sore uvula with white lesions that had been present since his surgical discharge. On postoperative day two, he was seen in our clinic stating that he had severe pain and difficulty with swallowing solids and liquids and felt the sensation of his uvula hitting one side when tilting his head, “like a wrecking ball.” On examination, we identified an elongated and swollen uvula with necrosis affecting a large portion of the uvular tip (Figure 3).

The airway was not compromised. Ibuprofen and magic mouthwash containing diphenhydramine, viscous lidocaine suspension, and aluminum hydroxide/magnesium hydroxide were recommended for treatment, and the patient was referred to the Ear/Nose/Throat (ENT) outpatient clinic due to his persistent symptoms. He was seen in the ENT clinic on postoperative day six and reported that his pain was improving. The patient was assessed and uvular elongation with a 5-mm extended fibrinous scabbing was noted. He was reassured that the expected course of improvement would be around two weeks, and the uvular elongation would likely continue despite healing.

## Discussion

Postoperative pharyngitis is a common adverse event with an incidence reported between 12% and 75% [7-9]. Both ETTs and LMAs are routinely used to secure the airway for patients under general anesthesia, and throat discomfort is typically attributed to the insertion of these devices [10]. The average duration of a sore throat postoperatively is around 16 hours [8]; therefore, persistent sore throat or foreign body sensation in the throat after surgery could indicate the rare complication of uvular necrosis. Patients with postoperative uvular necrosis can present with a sore throat, dysphagia, odynophagia, foreign body sensation, dyspnea, or gagging [6]. The condition is diagnosed through clinical examination of the uvula, which can appear elongated, swollen, and covered with white exudate at the necrotic tip [6].

The three patients in this report all underwent shoulder surgeries within a four-month window and were all male, varsity athletes. Previous findings have suggested that uvular necrosis is more likely to occur in males due to structural anatomy [6,11]. Only one prior report of uvular necrosis after shoulder arthroscopy has been documented, although the intubation instrumentation and patient positioning were not mentioned [12]. In our cases, uvular necrosis resulted from both ETT intubation and LMA placement. Reid et al. [6] found only 53 reported cases in the literature of post-operative uvular necrosis across various domains, including cardiac, abdominal, vascular, nasal, and orthopedic surgeries. ETTs were used in 68% of the uvular necrosis cases, whereas 4% of the cases were associated with LMAs. Uvular necrosis has also been reported following upper endoscopy [13], bronchoscopy [14], transesophageal echocardiography [15], and fiberoptic intubation [16]. Although the exact etiology of uvular necrosis is unknown, one cause can be attributed to the mechanical compression of the uvula by airway devices in the oropharynx that compromise uvular blood supply. Blind suctioning may also increase the risk of uvular injury [6].

Additionally, the cases in this report were performed in both the lateral decubitus and beach chair positions. It is unknown whether patient positioning affects the risk of uvular injury. A retrospective review of 10 patients diagnosed with post-operative uvular necrosis after undergoing general anesthesia found that every injury was noted in the supine position [11]. Further, one previous report described a patient who underwent laparoscopic donor nephrectomy with endotracheal intubation in the lateral decubitus position and subsequently developed uvular necrosis [17]. Interestingly, the athletes who developed uvular necrosis in this report all underwent shoulder surgery, although there is no known association between uvular injury and type of operation.

Pamnani et al. [11] found 10 patients with uvular necrosis within a three-year timeframe at an incidence rate of 0.034%. While we did not retrospectively review all procedures involving general anesthesia at our institution, the cases we reported all occurred within a relatively short, four-month window. As all three patients were varsity athletes, they had easy access to team physicians, athletic trainers, and physical therapists and were able to be examined in the clinic in a timely manner once their uvular symptoms began. Symptoms of uvular necrosis may not appear right away, and after surgery, patients are likely to focus their attention on pain from their operative extremity. Further, the majority of uvular necrosis cases resolve spontaneously within two weeks [6]. Most patients see their surgeon at a standard two-week postoperative visit, so it is possible that some patients with mild uvular necrosis symptoms could have complete resolution and go undetected.

Correctly recognizing this condition is important because in rare cases, uvular necrosis can cause airway obstruction, bleeding, or infection. Additionally, odynophagia and dysphagia can make nutrition and hydration difficult post-operatively. Treatment is largely conservative and focused on symptom management as symptoms typically self-resolve within days to weeks when the necrotic tip sloughs off [2,14]. In the literature, patients have been treated with steroids, anti-inflammatories, antihistamines, antibiotics, topical epinephrine, and lidocaine [2,11]. In select instances, surgery to excise the necrotic portion of the uvula has been suggested [3], although this is not well documented. Proposed methods to decrease the risk of uvular necrosis include placing ETTs to the side of the midline and avoiding blind suctioning [6].

## Conclusions

ETTs and LMAs are commonly used to secure the airway during surgical procedures involving general anesthesia. Uvular necrosis is a rare postoperative complication that can manifest from these instruments compressing and restricting blood flow to the uvula. We identified three cases of uvular necrosis following shoulder surgery in varsity athletes at our institution. Their symptoms included persistent sore throat, dysphagia, odynophagia, and foreign body sensation, with swelling and white exudate on the uvular tip. While symptoms typically self-resolve within two weeks, it is important to recognize the condition and risk factors because patients may benefit from reassurance and conservative treatment.

## References

[1] Arigliani M, Dolcemascolo V, Passone E, Vergine M, Cogo P (2016). Uvular trauma after laryngeal mask airway use. J Pediatr.

[2] Atkinson CJ, Rangasami J (2006). Uvula necrosis--an unusual cause of severe postoperative sore throat. Br J Anaesth.

[3] Calikapan GT, Karakus F (2008). Uvula necrosis after endotracheal intubation for rhinoplasty. Aesthetic Plast Surg.

[4] Commins DJ, Whittet H, Okoli UC, Ewart M (1994). Postintubation uvular necrosis. Anaesthesia.

[5] Emmett SR, Lloyd SD, Johnston MN (2012). Uvular trauma from a laryngeal mask. Br J Anaesth.

[6] Reid JW, Samy A, Jeremic G, Brookes J, Sowerby LJ (2020). Postoperative uvular necrosis: a case series and literature review. Laryngoscope.

[7] Christensen AM, Willemoes-Larsen H, Lundby L, Jakobsen KB (1994). Postoperative throat complaints after tracheal intubation. Br J Anaesth.

[8] Biro P, Seifert B, Pasch T (2005). Complaints of sore throat after tracheal intubation: a prospective evaluation. Eur J Anaesthesiol.

[9] Higgins PP, Chung F, Mezei G (2002). Postoperative sore throat after ambulatory surgery. Br J Anaesth.

[10] McHardy FE, Chung F (1999). Postoperative sore throat: cause, prevention and treatment. Anaesthesia.

[11] Pamnani A, Faggiani SL, Hood M, Kacker A, Gadalla F (2014). Uvular injury during the perioperative period in patients undergoing general anesthesia. Laryngoscope.

[12] Goldin M, Ji L (2013). Uvula necrosis, an atypical presentation of sore throat. J Emerg Med.

[13] Shores NJ, Bloomfeld RS (2009). Images in clinical medicine. Uvular necrosis after endoscopy. N Engl J Med.

[14] Sunio LK, Contractor TA, Chacon G (2011). Uvular necrosis as an unusual complication of bronchoscopy via the nasal approach. Respir Care.

[15] Nijjer S, Crean A, Li W, Swan L (2009). Uvular ulceration following transoesophageal echocardiography. BMJ Case Rep.

[16] Budde A, Parsons C, Eikermann M (2018). Uvula necrosis after fibreoptic intubation. Br J Anaesth.

[17] Chatterjee A, Kannaujia A, Paul M, Verma A (2018). Can lateral decubitus cause uvular necrosis after general anesthesia?. J Clin Anesth.

